# A Population Pharmacokinetic Model of Intravenous Dexmedetomidine for Mechanically Ventilated Children after Neurosurgery

**DOI:** 10.3390/jcm8101563

**Published:** 2019-10-01

**Authors:** In-Kyung Song, SoJeong Yi, Hyeong-Seok Lim, Ji-Hyun Lee, Eun-Hee Kim, Joo-Youn Cho, Min-Chang Kim, Jin-Tae Kim, Hee-Soo Kim

**Affiliations:** 1 Department of Anesthesiology and Pain Medicine, Asan Medical Center, University of Ulsan College of Medicine, Seoul 05505, Korea; goingridgo@gmail.com; 2 Department of Clinical Pharmacology and Therapeutics, Seoul National University College of Medicine and Hospital, Seoul 03080, Korea; sojeongyi83@gmail.com (S.Y.); joocho@snu.ac.kr (J.-Y.C.); mckim@snu.ac.kr (M.-C.K.); 3 Department of Clinical Pharmacology and Therapeutics, Asan Medical Center, University of Ulsan College of Medicine, Seoul 05505, Korea; mdhslim@gmail.com; 4 Department of Anesthesiology and Pain medicine, Seoul National University Hospital, Seoul National University College of Medicine, Seoul 03080, Korea; muslab@hanmail.net (J.-H.L.); beloveun@gmail.com (E.-H.K.); jintae73@gmail.com (J.-T.K.)

**Keywords:** child, deep sedation, dexmedetomidine, intensive care units, pharmacokinetics, preschool child

## Abstract

Dexmedetomidine is a selective alpha-2 adrenergic agonist with concurrent sedative and analgesic effects, and it is being increasingly used in pediatric anesthesia and intensive care. This study aimed to investigate the pharmacokinetics of intravenous dexmedetomidine in mechanically ventilated children in the intensive care unit (ICU) after neurosurgery. Pediatric patients aged 2–12 years, who were mechanically ventilated in ICU after neurosurgery, were allocated into a low-dose (*n* = 15) or high-dose (*n* = 14) group. The low-dose group received dexmedetomidine at a loading dose of 0.25 µg/kg for 10 min, followed by a maintenance dose of 0.25 µg/kg/h for 50 min, whereas the high-dose group received dexmedetomidine at a loading dose of 0.5 µg/kg for 10 min, followed by a maintenance dose of 0.5 µg/kg/h for 50 min. Serial blood samples were collected for a pharmacokinetic analysis up to 480 min after the end of the infusion. The sedative effect of dexmedetomidine was assessed using the Bispectral Index and University of Michigan Sedation Scale. Adverse reactions, electrocardiography findings, and vital signs were monitored for a safety assessment. A population pharmacokinetic analysis was performed using non-linear mixed effects modeling. Dexmedetomidine induced a moderate-to-deep degree of sedation during infusion in both groups. The pharmacokinetics of dexmedetomidine were best described by a two-compartment disposition model with first-order elimination kinetics. The parameters were standardized for a body weight of 70 kg using an allometric power model. The population estimates (95% confidence interval) per 70 kg body weight were as follows: clearance of 81.0 (72.9–90.9) L/h, central volume of distribution of 64.2 (50.6–81.0) L, intercompartment clearance of 116.4 (90.6–156.0) L/h, and peripheral volume of distribution of 167 (132–217) L. No serious adverse reactions or hemodynamic changes requiring the discontinuation of dexmedetomidine were observed. Dexmedetomidine had increased clearance and volume of distribution in mechanically ventilated children in ICU after neurosurgery, thereby indicating the need to adjust the dosage to obtain a target plasma concentration.

## 1. Introduction

Dexmedetomidine (Precedex^®^, Hospira, Lake Forest, IL, USA) is a selective alpha-2 adrenergic agonist with concurrent sedative and analgesic effects, only approved by the U.S. Food and Drug Administration for the sedation of mechanically ventilated adults in an intensive care unit (ICU) and the procedural sedation of non-intubated adults. In adults, the dosing regimen of dexmedetomidine comprises a loading dose of 1 µg/kg for 10 min, followed by a maintenance infusion of 0.2–1 µg/kg/h [1].

Currently, dexmedetomidine is not approved for use in children. However, dexmedetomidine is commonly applied in children as a premedication for anxiolysis, as a sedative for non-invasive or invasive procedures, and as an adjunct for analgesia [2]. Considering the unique properties of dexmedetomidine, such as minimal respiratory depression [3], it may be used in pediatric patients in the ICU as well as in the emergency and interventional rooms. Therefore, a significant increase in the use of dexmedetomidine is expected. Nevertheless, most studies performed on children are underpowered interventional studies or small observational studies of limited methodological quality [2]. Moreover, previous studies have used different dosages because the dosing regimen of dexmedetomidine in pediatric patients has not been established. The loading dose ranges from 0.05 to 6 µg/kg, whereas the maintenance dose ranges from 0.05 to 1.4 µg/kg/h [4,5,6,7,8,9,10,11].

According to the manufacturer’s prescribing information for dexmedetomidine, the dosage should be individualized and titrated to the desired clinical effect based on the patient’s body weight [1]. However, considering that the degree of maturation of various organs differs among children, over- or under-dosing may occur, which can lead to serious complications, side effects and a lack of expected therapeutic effects [11]. Therefore, the inter-individual differences affecting the pharmacokinetic (PK) profiles of dexmedetomidine in children must be identified to optimize the dosing regimen. To date, the PKs of dexmedetomidine in children have been investigated in some studies [4,5,6,7,8,9,10,11,12,13,14]. Nevertheless, information about the use of dexmedetomidine in children after neurosurgery is limited. Thus, the present study aimed to characterize the PKs and safety of intravenous dexmedetomidine in mechanically ventilated children in the ICU after neurosurgery.

## 2. Materials and Methods

### 2.1. Study Design and Participants

This randomized, patient-blinded trial was approved by the Seoul National University Hospital Institutional Review Board (3 June 2014, IRB No. 1403-073-564). After obtaining a written informed consent from all parents and a written assent from children older than 7 years, pediatric patients aged between 2 and 12 years, having an American Society of Anesthesiologists physical status classification of 1 or 2 and who were scheduled for neurosurgery and postoperative ventilator care for less than 4 h in the ICU, were enrolled prospectively. The exclusion criteria included patients with an altered mental status prior to surgery, a history of drug allergy and cardiovascular, hepatic, or renal disease, a body mass index ≥ 35 kg/m^2^, as well as those with hypovolemia, those on a chronic use of any medications, those undergoing hemodialysis, and those with a planned treatment for patient-controlled analgesia with opioid use. The study was registered at cris.nih.go.kr (KCT0001150). All procedures performed in this study were in accordance with the Declaration of Helsinki.

### 2.2. Study Protocol

The patients were stratified according to age: 10 in each age group (2–6, 6–9, and 9–12 years). The patients in each age group were randomly assigned to the low- or high-dose group by the Medical Research Collaborating Center of the Seoul National University Hospital. The randomization was sequenced into blocks of 2 and 4. The low-dose group received dexmedetomidine at a loading dose of 0.25 µg/kg for 10 min, followed by a maintenance dose of 0.25 µg/kg/h for 50 min. The high-dose group received dexmedetomidine at a loading dose of 0.5 µg/kg for 10 min, followed by a maintenance dose of 0.5 µg/kg/h for 50 min. The dosing regimen was based on previous studies conducted on the pediatric population receiving intravenous dexmedetomidine for ICU sedation [4,10].

An intravenous route was established before transporting the patient to the operating room and no premedication was administered. The standard monitoring in the operating room included electrocardiography, pulse oximetry (SpO_2_), non-invasive blood pressure, invasive arterial blood pressure, Bispectral Index™ (BIS; Covidien, Mansfield, MA, USA), train-of-four (TOF; neuromuscular transmission, S/5^TM^ Anesthesia Monitor, E-NMT module, Pediatric MechanoSensor, GE Healthcare, Datex-Ohmeda, Chicago, IL, USA), and esophageal temperature. All patients underwent an institutionalized general anesthetic procedure that included induction with 0.02 mg/kg of atropine, 2–3 mg/kg propofol, and 0.5–1 µg/kg of sufentanil; neuromuscular blockade with 0.6 mg/kg of rocuronium; and tracheal intubation, followed by mechanical ventilation with an air–oxygen mixture. During the maintenance of anesthesia, propofol was infused with the target-controlled infusion software (Asan pump, Bionet Co., Ltd., Seoul, Korea) using Kim’s model [15], and sufentanil was continuously administered starting from 0.5 µg/kg/h to maintain BIS values between 40 and 60. No additional neuromuscular blocking agents were allowed intraoperatively because of the electrophysiological monitoring during neurosurgery. The administration of sufentanil was discontinued 1 h prior to the end of surgery, and the propofol infusion was stopped at the end of the surgery. At the end of the surgery, all patients received an ultrasound-guided scalp nerve block with 0.25% ropivacaine [16].

After the surgery, all patients were transferred directly to the ICU without reversing the neuromuscular blockade. The intravenous infusion of dexmedetomidine was initiated in the ICU 30 min after arrival from the operating room. Once the patients arrived at the ICU, electrocardiography, vital signs including the mean blood pressure (MBP), heart rate (HR), SpO_2_, and respiratory rate (RR), and BIS were continuously monitored for all the patients and automatically recorded during the study period. The use of dexmedetomidine was discontinued after 60 min of infusion, and weaning from mechanical ventilation was attempted. The PKs of dexmedetomidine were evaluated up to 480 min after the end of the infusion and a safety assessment was conducted up to 24 h after the end of the infusion. During the infusion of dexmedetomidine, the administration of any other sedatives or analgesics was not allowed. After discontinuing the dexmedetomidine infusion, the patients were managed by the attending surgeons.

### 2.3. Blood Sampling and Drug Assays for PKs

One milliliter of arterial blood was sampled into ethylenediaminetetraacetic acid-containing tubes for the PK analysis just before the initiation of the infusion, 10, 30, and 60 min after the initiation of infusion and 15, 30, 60, 120, 240, and 480 min after the end of the infusion. All samples were centrifuged at 252× *g* for 10 min, and the plasma samples were stored at −70 °C until the assay.

The plasma concentration of dexmedetomidine was determined using a validated liquid chromatography/tandem mass spectrometry (LC-MS/MS) method with tolazoline as an internal standard [17,18]. In brief, dexmedetomidine and tolazoline were extracted from 100 μL of human plasma with methyl tert-butyl ether and 5 N of ammonium hydroxide. The samples were dried in a SpeedVac, reconstituted with 75 μL of a methanol:water mixture (1:1, *v*:*v*) with 0.1% formic acid, and injected into the LC-MS/MS (Agilent 1260 Infinity Binary LC (Agilent Technologies, Santa Clara, CA, USA) and API 4000 QTRAP system (AB Sciex, Framingham, MA, USA)). Chromatographic separation was achieved using the Luna CN column (2.1 × 100 mm, 3 µm, Phenomenex, Torrance, CA, USA) and distilled water and acetonitrile containing 0.1% formic acid (40:60, *v*/*v*) as the mobile phase. Dexmedetomidine and the internal standard were monitored in the positive ionization mode using the multiple reaction monitoring method with the transitions of *m*/*z* 201.16 → 95.03 and 161.11 → 91.10, respectively. The lower limit of quantification (LLOQ) for dexmedetomidine was 0.005 ng/mL, with a quantitation range of 0.005–2 ng/mL. The between-run coefficient of variation was <9.052%, and the accuracy was 91.63%–108.0%.

### 2.4. Efficacy and Safety Assessment

The sedative effect of dexmedetomidine was assessed using the BIS and University of Michigan Sedation Scale (UMSS) according to the time course [19,20,21,22]. The BIS and UMSS scores were measured consecutively after collecting the PK blood samples. The investigator categorized the UMSS score from 0 to 4: 0, awake and alert; 1, minimally sedated, tired/sleepy, appropriate response to verbal conversation and/or sound; 2, moderately sedated, somnolent/sleeping, easily aroused with light tactile stimulation or a simple verbal command; 3, deeply sedated, deep sleep, arousable only with significant physical stimulation; and 4, unarousable.

Adverse reactions, laboratory test results, electrocardiography findings, and vital signs were monitored for the safety assessment. The adverse reactions and laboratory results were followed-up until 24 h after the end of the infusion, and all the observed and reported adverse reactions were recorded. Vital signs, including the mean BP (MBP), HR, RR, and SpO_2_, were measured after collecting the PK blood samples and were considered abnormal when they exceeded preoperative baseline values by ± 20%. The relationship between adverse reactions and dexmedetomidine was graded as not related, probably not related, possibly related, probably related or definitely related by the investigator.

### 2.5. Population PK Analysis

The PK modeling was conducted using NONMEM^®^ (version 7.3; ICON Development Solutions, Ellicott City, MD, USA) with the ADVAN 6 subroutines and first-order conditional estimation with interaction.

To build the structural model, one-, two-, and three-compartment models were fitted to the dexmedetomidine plasma concentration data. The PK parameters of children (*p*) were standardized for a body weight (BW) of 70 kg using the allometric model as *p* = P_pop_ × (BW/70)^n^, where P_pop_ was the parameter of a person weighing 70 kg, and n was the allometric weight exponent, which was 0.75 for clearance and intercompartment clearance, and 1 for volume of distribution [23,24].

Exponential terms following a log-normal distribution were assumed for the description of the inter-individual variability in the PK parameters as P_i_ = θ × exp(η_i_), where P_i_ was the parameter of the i individual, θ was the typical population value of the parameter, and η_i_ was the inter-individual random effect assumed to have a mean of zero and a variance of ω^2^. An additive, constant coefficient of variation, and combined additive and proportional variance models were applied for the residual error during the model-building process.

The covariate effect on the PK model was assessed using a forward selection, in which a variable contributing to a significant decrease in the objective function value (OFV, decrease of at least 3.84 (α = 0.05)) was selected. Eight covariates (age, height, weight, body surface area [25], lean body mass [26], ideal BW [27], body mass index, and body fat percentage [28]) were evaluated using the exponential model (individual parameter = population parameter × {individual covariate value (median covariate value^−1^)}^θ^).

Plasma concentrations below the LLOQ (BLQ) before the first time point above the LLOQ were discarded, and in the elimination phase only the first data point of BLQ was substituted with LLOQ/2 (= 0.0025 ng/mL) and subsequent BLQ points were discarded [29].

The goodness-of-fit of each NONMEM analysis was investigated using plots of predicted plasma concentrations versus measured plasma concentrations and versus weighted residuals. The bootstrap resampling method was used to evaluate the stability and robustness of the final PK model. Resampling with a replacement generated 1000 bootstrap data sets, and the final population PK model was fitted repeatedly to each of them. Moreover, 95% confidence intervals for the final parameters were obtained from the bootstrap empirical posterior distribution. A visual predictive check (VPC) was used to evaluate the predictive performance of the final PK model. The observed data points were overlaid with the 5th, 50th, and 95th percentile curves of 1000 datasets simulated using the parameter estimates in the final model.

### 2.6. Statistics

A statistical analysis was performed using IBM^®^ SPSS^®^ Statistics 23 (SPSS Inc., IBM Corporation, Armonk, NY, USA). The continuous variables were analyzed using the Mann-Whitney U test, and the categorical variables were analyzed using the Fisher’s exact test. The data were presented as medians (IQR) or numbers (percentage). A *p* value of < 0.05 was considered statistically significant.

## 3. Results

### 3.1. Overall

From September 2014 to August 2015, among the 31 patients enrolled, 29 were randomly assigned to one of the two dexmedetomidine regimen groups. Two patients were excluded before allocation because of changes in the postoperative management plan. No significant group difference was observed in terms of the baseline and clinical characteristics (Table 1). The median (interquartile range, IQR) interval between the end of surgery (discontinuation of propofol) and the start of the dexmedetomidine infusion was 30 (30–31) min.

### 3.2. Efficacy and Safety

**Figure 1:**
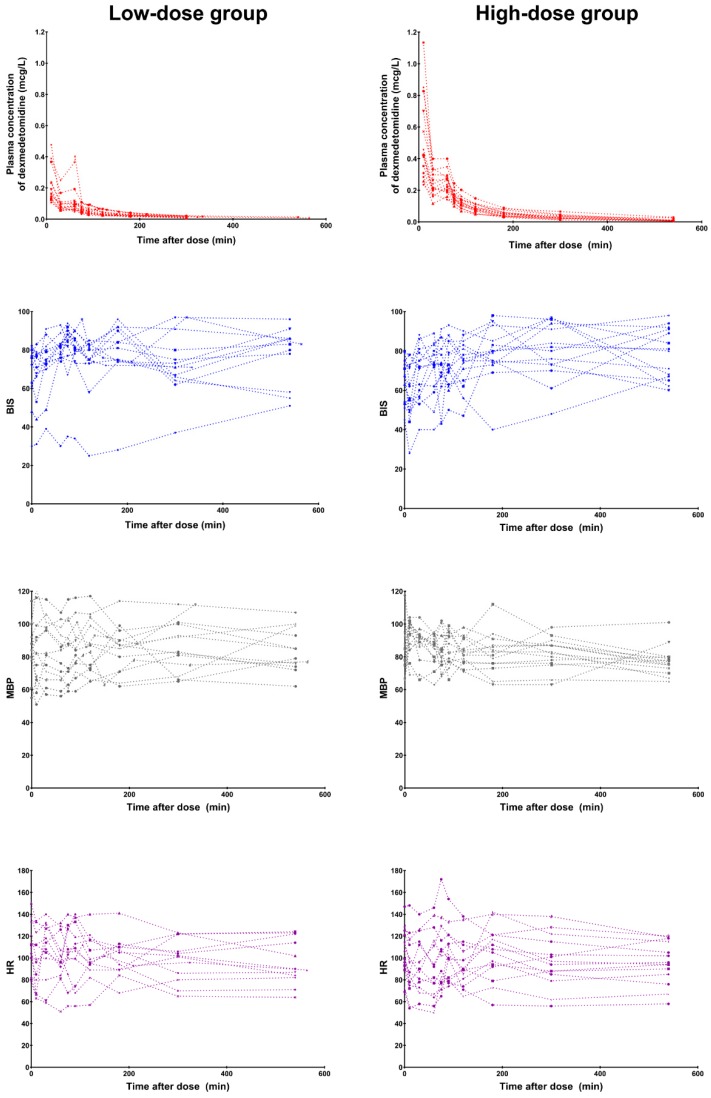
The observed plasma concentrations of dexmedetomidine (1st row), Bispectral Index (BIS, 2nd row), mean blood pressure (MBP, 3rd row), and heart rate (HR, 4th row) over time by dose group. Time after dose: time after starting the loading dose of dexmedetomidine.

Dexmedetomidine induced a moderate-to-deep degree of sedation during the infusion in both groups [median (IQR); BIS 76 (62–91) and UMSS 2 (0–3) in the low-dose group; BIS 73 (55–92) and UMSS 2 (1–3) in the high-dose group]. There were no differences in the median BIS (*p* = 0.650) and UMSS (*p* = 0.685) values between the groups. The time courses of the observed plasma concentrations of dexmedetomidine, BIS values, MBP, and HR in each group are presented in Figure 1.

Among the 29 participants who received dexmedetomidine via an intravenous infusion, no serious adverse reactions requiring the discontinuation of dexmedetomidine were observed. Overall, 11 (38%) patients presented with adverse reactions; however, the relationship between adverse reactions and dexmedetomidine was graded as probably not related. In detail, four patients (three from the low-dose group during and after infusion and one from the high-dose group during infusion) presented with tachycardia, two (one from each group) with bradycardia during and after infusion, and five (three from the low-dose group and two from the high-dose group) with both tachycardia and bradycardia during and after infusion. Furthermore, one patient from the low-dose group experienced hypertension during and after infusion. All patients who had adverse reactions did not require any intervention to resolve such events. No clinically significant abnormalities were observed in the laboratory test results.

### 3.3. Pharmacokinetics

Of the 264 plasma samples obtained from 29 participants, 19 were BLQ (<0.005 ng/mL), of which four were discarded and 15 were substituted with the LLOQ/2 for the population PK analysis. The time–concentration profiles of dexmedetomidine were best described using a two-compartment disposition model with first-order elimination kinetics. The final parameter estimates standardized for a BW of 70 kg using the allometric model are summarized in Table 2. No other covariates had a significant influence on the weight-adjusted PK parameters.

**Figure 2:**
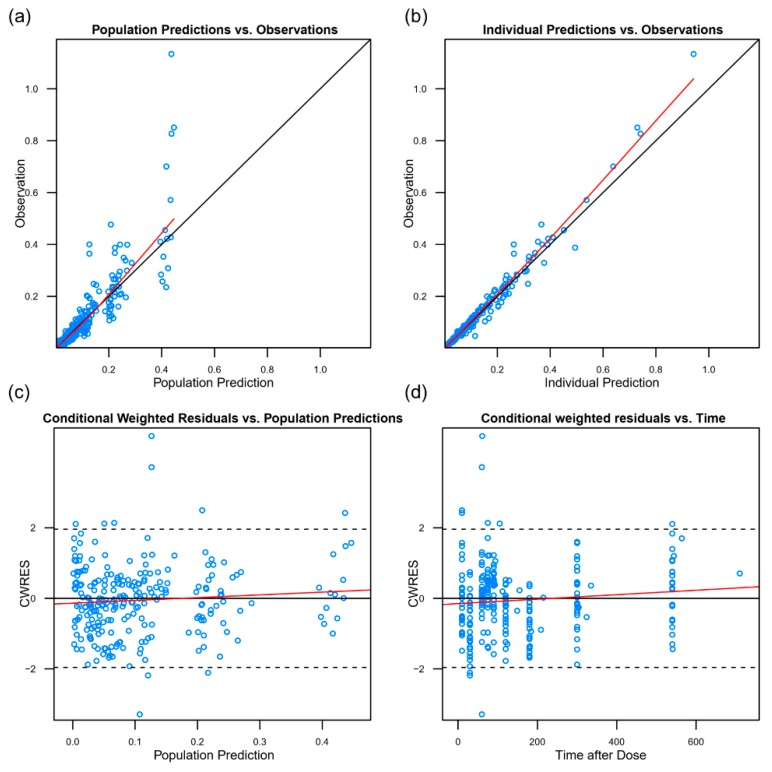
The goodness-of-fit plots for the population pharmacokinetic model. (**a**) The observed concentration vs. the population predicted concentration; (**b**) the observed concentration vs. the individual predicted concentration; (**c**) the individual weighted residual vs. the individual predicted concentration; (**d**) the weighted residuals vs. the time. The black lines = the line of identity in (**a**) and (**b**) and the line y = 0 in (**c**,**d**). The red line = smooth line with locally weighted regression.

**Figure 3:**
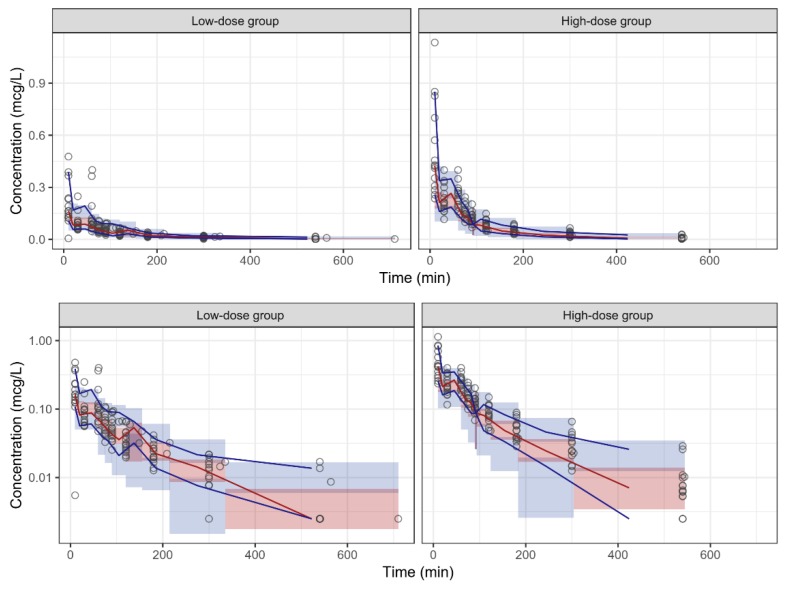
The linear (**upper**) and log-linear (**lower**) plots of the visual predictive check of the population pharmacokinetic model for dexmedetomidine by dose group. Circles = observed data; red solid line = median observed data; blue solid lines = 5th and 95th percentiles of the observed data; red area = 95% confidence intervals (CI) for the median simulated data; blue area = 95% CI for the 5th and 95th percentiles of the simulated data.

The goodness-of-fit plots indicated that the population PK model fitted well with the observed data (Figure 2). Most data were within the 5th and the 95th percentiles in a VPC, which indicated that the model had an acceptable predictive performance for dexmedetomidine PKs (Figure 3).

## 4. Discussion

In this study, we developed a population PK model of intravenous dexmedetomidine in a single population of patients aged 2–12 years who were under mechanical ventilation in the ICU after neurosurgery. The PK estimates of dexmedetomidine in pediatric patients, as previously reported in the literature, are summarized in Table S1. Similar to previous studies of children with the PK characteristics of dexmedetomidine using a two-compartment model [4,8,9,11,14,30], our data indicated an increased clearance and volume of distribution compared to those in the previous reports.

The population PK models are essential in determining drug dosage because they describe the behavior of a drug in a specific population and the variability expected between individuals and factors that explain this variability [14]. Children vary in terms of size, body composition, and maturity of organs, primarily due to age, and all these factors affect the PK behavior of a drug via the processes of input, distribution, and elimination [23]. Thus, the importance of an adequate PK model in children is emphasized. Considering the difficulty of performing PK studies on the pediatric population, our data may provide useful information about the use of dexmedetomidine in children. After an appropriate validation, our PK model can be implemented in daily clinical practice and can further help in developing a population pharmacokinetic–pharmacodynamic model for the use of intravenous dexmedetomidine in this population.

We applied the allometric power model, and the population PK parameters were scaled with the BW, which was the primary covariate that was used in our analysis to compare adult and pediatric estimates. There is a strong theoretical basis for using allometric scaling to account for the influence of the body size on the PK parameters, especially in children [31]. Apart for weight, other covariates did not have a significant influence on the weight-adjusted PK parameters. The PK model that was presented might be useful for individualized dosing and for the target-controlled infusion of dexmedetomidine in this population.

Dexmedetomidine clearance is mainly determined by the liver blood flow [32] because this drug is primarily metabolized in the liver via glucuronidation by uridine 5′-diphospho-glucuronosyl-′ transferase and via hydroxylation by cytochrome P450, with less than 5% of the drug remaining in the unchanged form afterward [33]. The clearance in this study, allometrically extrapolated to a 70-kg person, was higher than that of previous reports on children [8,9,11,14,30]. There are several possibilities for this result. The first is that our study participants were relatively older, with no critical illnesses such as hepatic or renal failure. Second, the general condition of the patients and changes in the hemodynamic variables could have affected the dexmedetomidine clearance. Third, although this is not clear yet, Korean children may have genetic variations regarding drug-metabolizing enzymes such as CYP2A6, which may affect the dexmedetomidine clearance. Chinese children showed a similar clearance, but showed a larger volume of distribution and a longer terminal half-life compared to Caucasians [8]. Further studies in children with different ethnicities is needed to solve this issue. Finally, the ontogeny of the major organ functions, body composition, endogenous functions that process drug transfer and disposition influence the drug disposition and effect [34].

Dexmedetomidine, a highly lipophilic drug, is extensively distributed, readily crosses the blood–brain barrier and penetrates extravascular sites, resulting in a large apparent volume of distribution [9]. Since 94% of dexmedetomidine is bound to plasma proteins, mainly albumin and α1-acid glycoprotein [33], a lower protein concentration will result in a higher unbound fraction and, correspondingly, in larger volumes of distribution. The estimated central volume of distribution in our patients was similar to that obtained from the pediatric population, ranging from 21.9 to 106.0 L per 70 kg [8,9,11,13,14,30].

In this study, we aimed to induce a moderate degree of sedation, assessed by BIS and UMSS, by administering dexmedetomidine, because the optimal sedation in the ICU has been described as a state in which the patient is somnolent, responsive to the environment but untroubled by it, and without excessive movements [35]. Therefore, if the desired effect of dexmedetomidine is to be changed, the dosage adjustment should be different.

According to the package insert for dexmedetomidine, the most common adverse reactions are hypotension and bradycardia, with the highest reported incidence rates being 56% and 42%, respectively. Data about the safety profile of dexmedetomidine in children are limited [36]. In two retrospective studies, hypotension, hypertension, and bradycardia were the most common adverse effects [37,38]. In our study, the types of adverse reactions were similar to those found in previous reports, and the incidence rates were lower in children than in adults. Furthermore, all adverse reactions were self-limiting and resolved without any treatment. Under close monitoring, dexmedetomidine is tolerable in mechanically ventilated children in the ICU.

The current study had several limitations. First, the external validation of the PK model using target-controlled infusion was not performed in a separate population. Second, although we did not administer any additional sedative or analgesic during the dexmedetomidine infusion, confounding drugs, such as the sub-therapeutic levels of anesthetics or analgesics, might have had some effects. Third, we only enrolled pediatric patients who received postoperative ventilator care and did not have severe comorbidities; therefore, our model may not be applicable to critically ill children in the ICU.

In conclusion, our population PK model of dexmedetomidine for mechanically ventilated children in the ICU after neurosurgery showed an increased clearance and volume of distribution, thereby indicating the need to adjust the dosage to obtain a target plasma concentration.

## Figures and Tables

**Table 1:** Patient characteristics and perioperative variables of the study participants. Values are the median (interquartile range) or number (percentage).

	Low-Dose Group (*n* = 15)	High-Dose Group (n = 14)
Age, years	8.0 (5.0–10.0)	7.0 (3.3–10.3)
Sex, boys	6 (40)	8 (57)
Height, cm	121.9 (108.0–136.0)	128.0 (104.0–138.8)
Weight, kg	22.0 (19.5–30.5)	23.0 (16.5–37.8)
Body surface area, m^2^	0.9 (0.8–1.0)	0.9 (0.7–1.2)
Lean body mass, kg	18.2 (14.4–24.4)	21.5 (12.3–30.2)
Ideal body weight, kg	23.5 (17.8–31.1)	26.5 (16.4–32.9)
Body mass index, kg/m^2^	16.1 (13.8–17.6)	16.2 (15.1–19.7)
Body fat percentage, %	20 (17–22)	20 (17–23)
**Types of operation**		
Craniotomy & tumor removal	12 (79)	14 (100)
Encephaloduroarteriosynangiosis	1 (7)	0 (0)
Foramen magnum decompression	1 (7)	0 (0)
Endoscopic transsphenoidal surgery	1 (7)	0 (0)
Duration of operation, min	265 (240–300)	295 (268–321)
**Laboratory results**		
Plasma albumin, g/dL	4.4 (4.3–4.4)	4.5 (4.1–4.6)
Plasma bilirubin, mg/dL	0.5 (0.4–0.6)	0.5 (0.3–0.5)
Plasma creatinine, mg/dL	0.41 (0.32–0.45)	0.45 (0.32–0.52)

**Table 2:** Parameter estimates and standard error in the final population pharmacokinetic model.

Parameters	Estimate	RSE (%) ^a^	Bootstrap Median (95% Confidence Interval) ^b^
**Structural model**			
Allometric clearance of central compartment ^c^ (CL_pop_, L/h)	81.0	5.5	81.1 (72.9–90.9)
Allometric volume of central compartment ^c^ (V1_pop_, L)	64.2	12.6	63.7 (50.6–81.0)
Allometric clearance of peripheral compartment ^c^ (Q_pop_, L/h)	116.4	13.1	119.2 (90.6–156.0)
Allometric volume of peripheral compartment ^c^ (V2_pop_, L)	167	12.5	167 (132–217)
**Inter-individual variability**			
CL (CV%)	27.1	26.6	26.4 (18.5–34.2)
V1 (CV%)	60.0	27.6	57.4 (41.8–75.0)
Q (CV%)	46.7	55.0	44.7 (12.7–65.7)
V2 (CV%)	60.7	36.3	59.5 (39.0–81.9)
**Residual error**			
Additive error (µg/L)	0.0227	81.5	0.0245 (0.0179–0.0515)
Proportional error (%)	42.7	14.2	42.0 (33.4–47.6)

^a^ RSE, the relative standard error was estimated using the following equation; RSE (%) = 100 × standard error/parameter estimate. ^b^ 95% confidence interval estimated by applying the final population pharmacokinetic model to 1000 resampled dataset. ^c^ Allometric weight-normalized model (WT = body weight in kg): CL = CL_pop_ × (WT/70)^0.75^; V1 = V1_pop_ × (WT/70); Q = CL_pop_ × (WT/70)^0.75^; V2 = V2_pop_ × (WT/70).

## Supplementary Materials

The following are available online at https://www.mdpi.com/2077-0383/8/10/1563/s1, Table S1: Overview of previously published pharmacokinetic estimates of dexmedetomidine in the pediatric population, standardized for a body weight of 70 kg using the allometric model.

## References

[1] PRECEDEX—Dexmedetomidine Hydrochloride Injection, Solution. http://labeling.pfizer.com/ShowLabeling.aspx?id=4404.

[2] Plambech M.Z., Afshari A. (2015). Dexmedetomidine in the pediatric population: A review. Minerva Anestesiol..

[3] Mahmoud M., Mason K.P. (2015). Dexmedetomidine: Review, update, and future considerations of paediatric perioperative and periprocedural applications and limitations. Br. J. Anaesth..

[4] Diaz S.M., Rodarte A., Foley J., Capparelli E.V. (2007). Pharmacokinetics of dexmedetomidine in postsurgical pediatric intensive care unit patients: Preliminary study. Pediatr. Crit. Care Med..

[5] Chrysostomou C., Schulman S.R., Herrera Castellanos M., Cofer B.E., Mitra S., da Rocha M.G., Wisemandle W.A., Gramlich L. (2014). A phase II/III, multicenter, safety, efficacy, and pharmacokinetic study of dexmedetomidine in preterm and term neonates. J. Pediatr..

[6] Su F., Gastonguay M.R., Nicolson S.C., DiLiberto M., Ocampo-Pelland A., Zuppa A.F. (2016). Dexmedetomidine Pharmacology in Neonates and Infants After Open Heart Surgery. Anesth. Analg..

[7] Vilo S., Rautiainen P., Kaisti K., Aantaa R., Scheinin M., Manner T., Olkkola K.T. (2008). Pharmacokinetics of intravenous dexmedetomidine in children under 11 yr of age. Br. J. Anaesth..

[8] Liu H.C., Lian Q.Q., Wu F.F., Wang C.Y., Sun W., Zheng L.D., Schuttler J., Ihmsen H. (2017). Population Pharmacokinetics of Dexmedetomidine After Short Intravenous Infusion in Chinese Children. Eur. J. Drug Metab. Pharmacokinet.

[9] Potts A.L., Anderson B.J., Warman G.R., Lerman J., Diaz S.M., Vilo S. (2009). Dexmedetomidine pharmacokinetics in pediatric intensive care--a pooled analysis. Paediatr. Anaesth..

[10] Su F., Nicolson S.C., Gastonguay M.R., Barrett J.S., Adamson P.C., Kang D.S., Godinez R.I., Zuppa A.F. (2010). Population pharmacokinetics of dexmedetomidine in infants after open heart surgery. Anesth. Analg..

[11] Wiczling P., Bartkowska-Sniatkowska A., Szerkus O., Siluk D., Rosada-Kurasinska J., Warzybok J., Borsuk A., Kaliszan R., Grzeskowiak E., Bienert A. (2016). The pharmacokinetics of dexmedetomidine during long-term infusion in critically ill pediatric patients. A Bayesian approach with informative priors. J. Pharmacokinet Pharmacodyn..

[12] Petroz G.C., Sikich N., James M., van Dyk H., Shafer S.L., Schily M., Lerman J. (2006). A phase I, two-center study of the pharmacokinetics and pharmacodynamics of dexmedetomidine in children. Anesthesiology.

[13] Greenberg R.G., Wu H., Laughon M., Capparelli E., Rowe S., Zimmerman K.O., Smith P.B., Cohen-Wolkowiez M. (2017). Population Pharmacokinetics of Dexmedetomidine in Infants. J. Clin. Pharmacol..

[14] Perez-Guille M.G., Toledo-Lopez A., Rivera-Espinosa L., Alemon-Medina R., Murata C., Lares-Asseff I., Chavez-Pacheco J.L., Gomez-Garduno J., Zamora Gutierrez A.L., Orozco-Galicia C. (2018). Population Pharmacokinetics and Pharmacodynamics of Dexmedetomidine in Children Undergoing Ambulatory Surgery. Anesth. Analg..

[15] Choi B.M., Lee H.G., Byon H.J., Lee S.H., Lee E.K., Kim H.S., Noh G.J. (2015). Population pharmacokinetic and pharmacodynamic model of propofol externally validated in children. J. Pharmacokinet Pharmacodyn..

[16] Shah R.D., Suresh S. (2013). Applications of regional anaesthesia in paediatrics. Br. J. Anaesth..

[17] Lee J.I., Su F., Shi H., Zuppa A.F. (2007). Sensitive and specific liquid chromatography-tandem mass spectrometric method for the quantitation of dexmedetomidine in pediatric plasma. J. Chromatogr. B Analyt. Technol. Biomed. Life Sci..

[18] Li W., Zhang Z., Wu L., Tian Y., Feng S., Chen Y. (2009). Determination of dexmedetomidine in human plasma using high performance liquid chromatography coupled with tandem mass spectrometric detection: Application to a pharmacokinetic study. J. Pharm. Biomed. Anal..

[19] Sadhasivam S., Ganesh A., Robison A., Kaye R., Watcha M.F. (2006). Validation of the bispectral index monitor for measuring the depth of sedation in children. Anesth. Analg..

[20] McDermott N.B., VanSickle T., Motas D., Friesen R.H. (2003). Validation of the bispectral index monitor during conscious and deep sedation in children. Anesth. Analg..

[21] Malviya S., Voepel-Lewis T., Tait A.R., Watcha M.F., Sadhasivam S., Friesen R.H. (2007). Effect of age and sedative agent on the accuracy of bispectral index in detecting depth of sedation in children. Pediatrics.

[22] Sciusco A., Standing J.F., Sheng Y., Raimondo P., Cinnella G., Dambrosio M. (2017). Effect of age on the performance of bispectral and entropy indices during sevoflurane pediatric anesthesia: A pharmacometric study. Paediatr. Anaesth..

[23] Holford N., Heo Y.A., Anderson B. (2013). A pharmacokinetic standard for babies and adults. J. Pharm. Sci..

[24] Germovsek E., Barker C.I., Sharland M., Standing J.F. (2017). Scaling clearance in paediatric pharmacokinetics: All models are wrong, which are useful?. Br. J. Clin. Pharmacol..

[25] (1987). Simplified Calculation of Body-Surface Area. N. Eng. J. Med..

[26] Hallynck T.H., Soep H.H., Thomis J.A., Boelaert J., Daneels R., Dettli L. (1981). Should clearance be normalised to body surface or to lean body mass?. Br. J. Clin. Pharmacol..

[27] Traub S.L., Johnson C.E. (1980). Comparison of methods of estimating creatinine clearance in children. Am. J. Hosp. Pharm..

[28] Deurenberg P., Weststrate J.A., Seidell J.C. (1991). Body mass index as a measure of body fatness: Age- and sex-specific prediction formulas. Br. J. Nutr..

[29] Keizer R.J., Jansen R.S., Rosing H., Thijssen B., Beijnen J.H., Schellens J.H., Huitema A.D. (2015). Incorporation of concentration data below the limit of quantification in population pharmacokinetic analyses. Pharmacol. Res. Perspect..

[30] Potts A.L., Warman G.R., Anderson B.J. (2008). Dexmedetomidine disposition in children: A population analysis. Paediatr. Anaesth..

[31] Anderson B.J., Holford N.H. (2011). Tips and traps analyzing pediatric PK data. Paediatr. Anaesth..

[32] Dutta S., Lal R., Karol M.D., Cohen T., Ebert T. (2000). Influence of cardiac output on dexmedetomidine pharmacokinetics. J. Pharm. Sci..

[33] Li A., Yuen V.M., Goulay-Dufay S., Kwok P.C. (2016). Pharmacokinetics and pharmacodynamics of dexmedetomidine. Drug Dev. Ind. Pharm..

[34] Van den Anker J., Reed M.D., Allegaert K., Kearns G.L. (2018). Developmental Changes in Pharmacokinetics and Pharmacodynamics. J. Clin. Pharmacol..

[35] Harris J., Ramelet A.S., van Dijk M., Pokorna P., Wielenga J., Tume L., Tibboel D., Ista E. (2016). Clinical recommendations for pain, sedation, withdrawal and delirium assessment in critically ill infants and children: An ESPNIC position statement for healthcare professionals. Intensive Care Med..

[36] Su F., Hammer G.B. (2011). Dexmedetomidine: Pediatric pharmacology, clinical uses and safety. Expert Opin. Drug Saf..

[37] Carroll C.L., Krieger D., Campbell M., Fisher D.G., Comeau L.L., Zucker A.R. (2008). Use of dexmedetomidine for sedation of children hospitalized in the intensive care unit. J. Hosp. Med..

[38] Mason K.P., Zurakowski D., Zgleszewski S.E., Robson C.D., Carrier M., Hickey P.R., Dinardo J.A. (2008). High dose dexmedetomidine as the sole sedative for pediatric MRI. Paediatr. Anaesth..

