# Supplementary material for: A Population Pharmacokinetic Model of Intravenous Dexmedetomidine for Mechanically Ventilated Children after Neurosurgery
Source: J Clin Med. 2019 Oct 1;8(10):1563. doi: 10.3390/jcm8101563 (PMC6832515; doi:10.3390/jcm8101563)
Supplement: Supplementary file 1 [file jcm-08-01563-s001.pdf]

Table S1 Overview of previously published pharmacokinetic estimates of dexmedetomidine in the pediatric population, standardized for a body weight of 70 kg using the allometric model. Values are median (range) or mean ± SD.

Study	N	Age<br>year	Weight<br>kg	Dosing regimen	Population	Model	Pharmacokinetic parameters<br>CL (L/h)	V1 (L)	Q (L/h)	V2 (L)
This study	29	7.0 (2.0–12.0)	22.0 (11.0–58.0)	0.25, 0.5 μg/kg for 10 min followed by 0.25, 0.5 μg/kg/h	ICU	2-comp	81.0	64.2	116.4	167.0
Potts (2008)	45	3.4 (0.0–14))	15.1 (3.1–58.9)	1–4 μg/kg for 10 min	ICU	2-comp	39.2	36.9	69.9	68.2
Potts (2009)	95	3.8 (0.0–14.4)	16.1 (3.1–58.9)	1–6 μg/kg for 5–10 min or 0.2 μg/kg/h	Pooled analysis	2-comp	42.1	56.3	78.3	69
Wiczling (2016)	38	5.8 (0.1–15.7)	18.5 (4.7–60)	0.8 μg/kg/h	ICU	2-comp	41.6	56.8	52.0	70.4
Liu (2017)	36	3.0 (1–9)	14.3 (10.0–27.0)	1.0, 1.5, 2.0 μg/kg for 10 min	Surgery	2-comp	36.2	84.3	82.8	114.0
Greenberg (2017)	20	0.1 (0.0–0.6)	4.0 (2.0–6.0)	1 μg/kg/h continuous infusion with 0.5 μg/kg bolus as needed	ICU	1-comp	48.2	106.0		
Pérez-Guillé	30	11 ± 5	43 ± 19	0.7 μg/kg for 10–15 min	Surgery	2-comp	20.8	21.9	75.8	81.2

CL, clearance of central compartment; V1, volume of distribution of central compartment; Q, clearance of peripheral compartment; V2, volume of distribution of peripheral compartment; ICU, intensive care unit.
